# Multiscale Ultraviolet/Visible Absorption Spectroelectrochemistry in Parallel Configuration

**DOI:** 10.1021/acs.analchem.6c01563

**Published:** 2026-06-19

**Authors:** Maria Huidobro, Martin Perez-Estebanez, Aranzazu Heras, Alvaro Colina

**Affiliations:** Department of Chemistry, 16725 Universidad de Burgos, Pza. Misael Bañuelos s/n, E-09001 Burgos, Spain

## Abstract

Ensuring good quality UV/vis absorption measurements is crucial for obtaining reliable information during spectroelectrochemistry (UV/vis-SEC) experiments. One important factor in ensuring this quality is the initial value of the detected light, I_0_, which is necessary to define the absorbance (A = −log­(I/I_0_)). Low values of I_0_ or I can significantly alter the shape of the UV/vis spectra, particularly during the evolution of species with high absorption coefficients. In this work, we developed a new SEC technique, denoted as multiscale UV/vis-SEC, which performs simultaneous optical measurements at different scales, using a dual path length cell, to describe with high reliability the evolution of the reactants and products involved in the electrode process.

## Graphical abstract

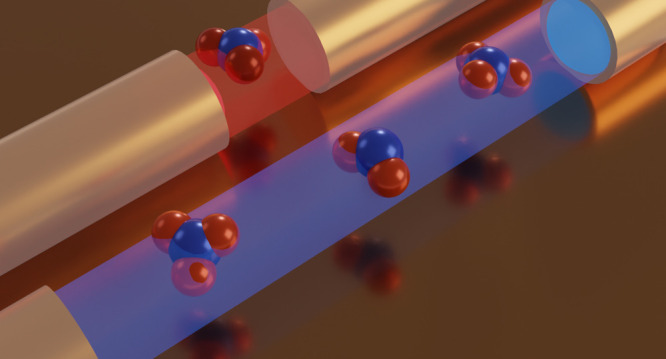

## Introduction

Spectroelectrochemistry (SEC) is a set of analytical techniques that provide complementary information about an electrochemical system from two different perspectives by simultaneously combining spectroscopic and electrochemical measurements within a single experiment. Among them, UV/vis absorption spectroelectrochemistry (UV/vis-SEC) stands out for its ability to elucidate reaction mechanisms, study diffusive processes, characterize electrochromic materials or perform quantitative analysis.^1–4^ UV/vis-SEC can be implemented in two different configurations, depending on the direction of the light beam sampling the electrode/solution interface: normal and parallel configurations.^5,6^ For the study of solution processes, the parallel configuration is the most interesting. In the parallel arrangement, the light beam passes parallel to the working electrode (WE) surface, sampling only the diffusion layer adjacent to it and supplying information about all changes related to the compounds in solution. This configuration is characterized by its great sensitivity, thanks to its long optical pathway, which can equal the length of the WE, allowing the detection of low-concentration species in solution, making it well-suitable for quantitative analysis and the study of reaction processes.^5^

Time-resolved (TR) operando UV/vis-SEC measurements involve recording the evolution of the entire UV/vis spectrum during an electrochemical experiment. Information about the species consumed or generated during the electrochemical process can be obtained from the evolution of the absorption bands. Working under semi-infinite diffusion conditions, the Lambert–Beer law (eq 1) can be applied, but considering the diffusion profile of the compounds generated or consumed.^7^

$$A=-\log\left(\frac{I}{I_{0}}\right)=\varepsilon_{\lambda }\times l\times C$$ 1

where A is the absorbance, I_0_ is the reference intensity of the incident light beam, I is the intensity of the transmitted light beam during the experiment, ε_λ_ is the molar extinction coefficient of the molecule at a specific wavelength, l is the optical path length, and C is the molar concentration of the molecule under study.

As is well-known, some limitations of Lambert–Beer law must be addressed when interpreting spectra recorded in UV/vis-SEC experiments. The first aspect to consider is the relationship between I_0_ and I. At very low analyte concentrations or for compounds with very low ε_λ_ values, the difference between I and I_0_ can be minimal, resulting in an I/I_0_ ratio of almost 1, leading to a measured absorbance close to 0. In such cases, the S/N ratio in the absorption bands measured across the entire spectral range is remarkably low, hindering spectral changes. Conversely, at very high analyte concentrations or for compounds with very high ε_λ_, I value can be very low because a higher proportion of the incident light is absorbed. Consequently, the difference between I and I_0_ is significant, with I/I_0_ approaching 0, resulting in negligible light transmission. These limitations of Lambert–Beer law, including the difference between ε_λ_^,^ can lead to problems that may be associated with the detection of artifacts, including ″false″ absorption bands, unexpected blue-shifting of absorption bands, or deviations from Lambert–Beer law (eq 1).^8–10^ It is crucial to take special care to differentiate the characteristic changes of a chemical system from artifacts in the absorption spectra.

Spectrophotometers used in TR-UV/vis-SEC are based on array detectors, allowing the simultaneous acquisition of spectra across a broad wavelength range.^10,11^ The experiments presented in this work cover the spectral range from 205 to 990 nm, including both the UV and visible regions. Bands in the UV region usually tend to have higher ε_λ_ values than those in the visible one. In some cases, the difference in the ε_λ_ values can be too high, making it impossible to obtain simultaneous and valuable information in both the UV and visible regions. In electrochemical processes, this is a critical issue because the absorption bands cannot be related to the same molecule, as the reactants and products can absorb both in the UV and/or visible regions, with concentration values that change during the SEC experiment, complicating the analysis. If there is a high difference in the intensity (I) of the bands in the two spectral regions, and assuming, for example, that the UV band is much higher than the visible one, the election of a long optical pathway would not allow the measurement of light in the UV region because I/I_0_ values would be close to 0, and only information about the visible region could be obtained. In contrast, a short optical pathway would allow measurements in the UV region, but the detection of the visible band could be compromised, resulting in ill-defined signals and absorbance values close to 0.

The quality of the spectroscopic measurements is crucial for obtaining reliable UV/vis-SEC information. For many years, SEC has been performed in the visible region of the spectrum, but advances in optics have allowed easy measurements in the UV region. Typically, most molecules absorb in the UV region of the spectrum, providing valuable information to follow the charge transfer process, as the electrochemical reaction helps to separate the spectra of the different compounds involved in it.^12^ As stated above, the ε_λ_ of molecules is typically higher in the UV region than in the visible region. Therefore, the selection of the best optical pathway to perform UV/vis-SEC measurements in a parallel configuration should reach a compromise solution when molecules have ε_λ_ values that differ significantly in the UV and visible regions and the researcher wants to obtain reliable information in the two spectral regions. Even with high-quality spectrophotometers and considering the limitations of Lambert–Beer law, in many cases, a single scale of measurement cannot be used to obtain reliable information. In such situations, performing a multiscale experiment could be much more useful for extracting all optical information in a single experiment.

Therefore, in this work, a new approach to work in UV/vis-SEC in a parallel configuration is proposed. The new setup allows us to select two different optical path lengths because the optical pathway is a scalable and controllable parameter, and perform absorptometric measurements simultaneously and in a single experiment at two different scales, obtaining more accurate and reliable information on the electrode process. This innovative approach is denoted as multiscale UV/vis-SEC, which provides control over different spectral regions without modifying the fundamental experimental setup by using a dual path length SEC cell.

The term “multiscale” was selected because it is primarily used in the fields of modeling and materials science to denote the analysis of complex chemical or physical systems across different scales of organization.^13,14^ This approach provides a complete picture of the changes in the absorption bands of the chemical system in the whole spectrum, even for compounds with very different molar absorption coefficients or concentrations, which would not have been possible for two independent experiments as reproducibility is not always guaranteed.

## Experimental Section

### Reagents and Materials

Ferrocenemethanol (FcMeOH, 97%, Sigma-Aldrich), potassium chloride (KCl, >99%, Acros Organics), potassium hydroxide (KOH, EMSURE, Sigma-Aldrich), potassium nitrate (KNO_3_, > 99%, Sigma-Aldrich) were used as provided without further purification. All solutions were prepared using ultrapure deionized water obtained from a Milli-Q SQ 2Series water purification system (Merck Millipore) with a resistivity of 18.2 MΩ·cm at 25 °C.

### Electrochemical Setup

A three-electrode system was used to perform all the experiments. A copper disk (3.18 mm diameter, 99.99% Alfa Aesar) embedded in Teflon or a glassy carbon (GC, 3 mm diameter, CHI instruments) was used as the working electrode (WE), a platinum wire was used as the counter electrode (CE), and a homemade Ag/AgCl (3 M KCl) or a reversible hydrogen electrode (RHE) was used as the reference electrode (RE). The WE was polished before use with 0.05 μm alumina and cleaned in deionized water under sonication.

### Instrumentation

UV/vis-SEC experiments were performed using two customized SPELEC instruments (Figure S1, Metrohm-DropSens). DropView SPELEC software (Metrohm-DropSens) was used to control and synchronize the instrument.

### Multiscale UV/Vis-SEC Cell

The multiscale UV/vis-SEC cell (Figure 1 and Figure S2) consisted of two bodies fabricated by 3D printing (Photon Mono SE, ANYCUBIC). The bottom body was designed to connect four 100 μm optical fibers (Ocean Optics) to achieve two different optical path lengths in the parallel arrangement. Therefore, the beam size was 100 μm because it is limited by the optical fiber core. The actual position of the beam was approximately at 30 μm away from the WE because of the cladding of the optical fibers.^15^ Thus, the light beam interrogates the diffusion layer close to the electrode. When the products of the reaction diffuse at a distance of the WE longer than 130 μm, the optical response can achieve a stationary response reaching a maximum of absorbance.^16^

**Figure 1:**
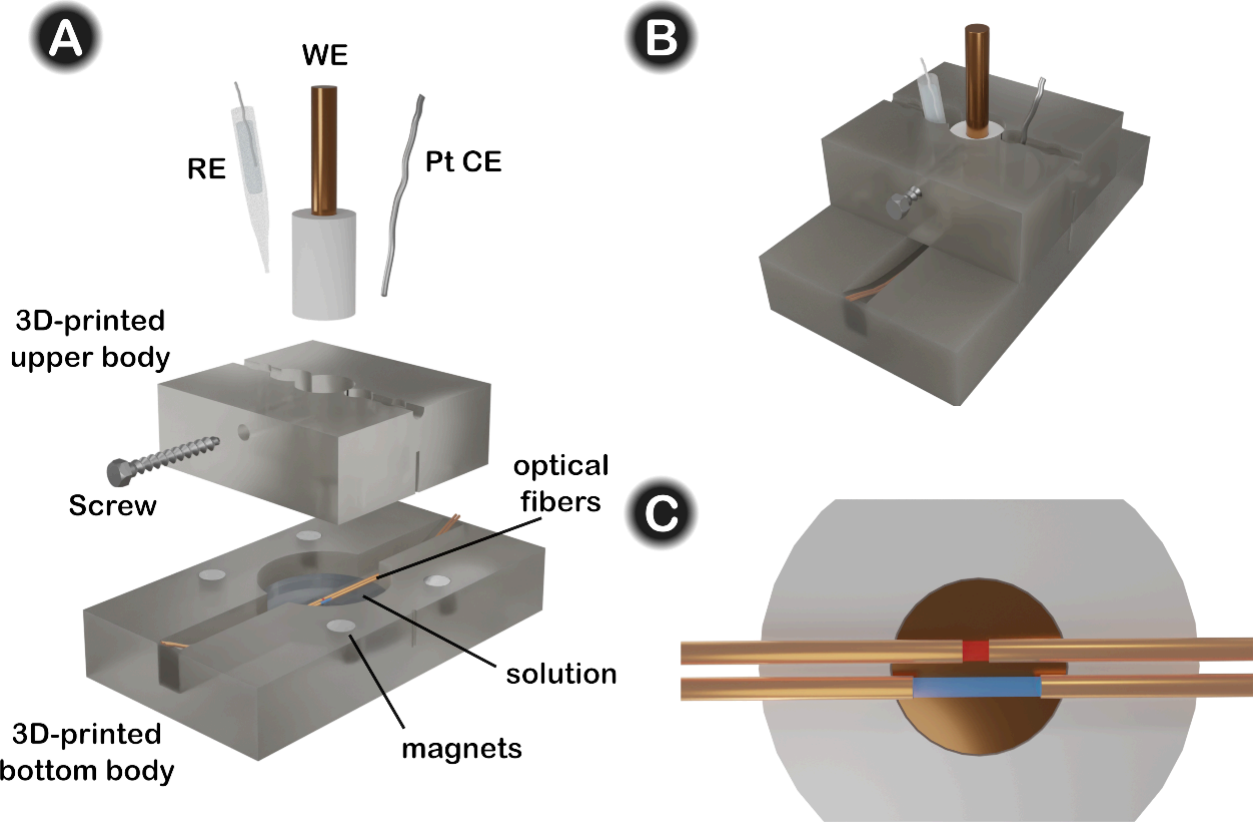
Schematic of the multiscale-UV/vis-SEC cell developed (A) disassembled; (B) assembled. (C) Schematic of the position of the two pairs of optical fibers with respect to the WE, where the two optical path lengths can be observed. Light was conducted in the opposite direction in each pair of optical fibers to avoid optical interference.

The bottom body also contains a pool for the solution with four steps to fix the optical fibers at different distances (Figure S2B,C). The distance between each pair of steps can be printed at different positions to better control the length of the two optical pathways. Four magnets were placed in each body to ease the assembly of both bodies. The upper piece has three holes for the three electrodes: WE, RE, and CE. Thus, the WE was inserted through the central hole, placing it just over the space between the two pairs of optical fibers (Figure 1C). To fix the position of the WE, a screw was inserted perpendicularly into the upper body. The RE and CE were inserted into the lateral holes of the upper body of the cell.

The two different optical path lengths allowed us to simultaneously record the evolution of the UV/vis absorption spectra at two different scales. The distances between each pair of optical fibers (optical path lengths used) were 2.9 mm for the longest pathway (lpw) and 0.6 mm for the shortest one (spw), resulting in a d_lpw_/d_spw_ ratio of 4.8. These path lengths can be changed according to the requirements or characteristics of the studied system.

Many UV/vis-SEC cells have been specifically developed for screen-printed electrodes^3^ or for electrode systems in which the three-electrode system is located on the same plane;^16^ however, few are designed to work in a parallel configuration with disk electrodes, which are commonly used by many researchers. It is noteworthy that the multiscale UV/vis-SEC cell proposed here facilitates measurements on disk electrodes to enable simple UV/vis-SEC measurements in the parallel configuration.

A key challenge in developing SEC cells is selecting chemically inert materials to fix the optical fibers which do not react or degrade under experimental conditions. In this work, Wax W (Apiezon) was used to fix the fibers to the bottom body of the cell because of its stability under extreme pH conditions, avoiding changes in the optical fiber position.

## Results and Discussion

### Validation of the Multiscale UV/Vis-SEC Cell

FcMeOH has been widely used as a redox probe in UV/vis-SEC experiments, because it is a reversible electrochemical system and the oxidation product displays two intense, overlapping absorption bands in the UV region, at 282 and 255 nm, and a very low intensity absorption band in the visible region, centered around at 625 nm.^16^ Cyclic voltammetry (CV) was performed to validate the SEC cell, using a GC-WE, an Ag/AgCl (3 M KCl) RE and a Pt-CE. Figure 2A shows the CV of FcMeOH in 0.1 M KCl performed between 0.00 V and +0.25 at 0.02 V·s^–1^, starting at 0.00 V in the anodic direction. The oxidation current increased by approximately +0.09 V, indicating the conversion of Fe­(II) to Fe­(III), leading to the formation of the FcMeOH^+^ cation. The oxidation process was limited by diffusion, with a maximum current at +0.18 V. In the reverse cathodic scan, a reduction current occurs owing to the opposite reaction, where the reduction of Fe­(III) to Fe­(II) regenerates FcMeOH.

**Figure 2:**
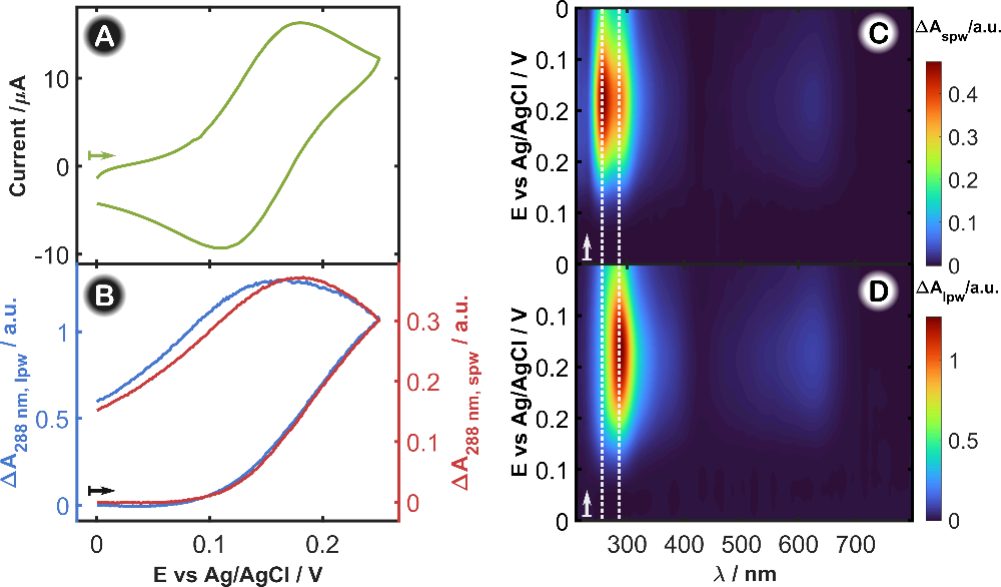
(A) CV and (B) CVAs at 288 nm for long (blue) and short (red) optical pathways of a SEC experiment. Contour plots showing the evolution of the absorption spectra for (C) short and (D) long optical pathways. Experimental conditions: 2.5 mM FcMeOH solution in 0.1 M KCl, CV from 0.00 V to +0.25 at 0.02 V·s^–1^.

UV/vis-SEC measurements were simultaneously recorded during the CV, and the evolution of absorbance at the two path lengths is shown in Figure 2C,D. In both optical path lengths, two absorption bands emerged coinciding with the oxidation of FcMeOH, one at 625 nm and the other in the UV range. In the visible region, there are notable similarities between the two absorption signals (Figure S3A). The main difference is the intensity of the absorbance band peaking at 625 nm, as shown in the cyclic voltabsorptograms (CVA, Figure S3C). The A_lpw_/A_spw_ absorbance ratio at 625 nm at the maximum value (+0.174 V in the backward scan) is 4.78, which is very close to the d_lpw_/d_spw_ ratio of 4.8. This value indicates that the same process was observed for the two optical path lengths in the visible region.

However, the UV region displays a significant discrepancy between the two optical path lengths (Figure 2C,D). For the longest optical path length, only a single absorption band was observed, which shifted between 282 and 290 nm during the oxidation/reduction process. Nevertheless, the shortest optical path length exhibited two overlapping absorption bands: one at 288 nm and another at 255 nm (inset, Figure S3A), with no appreciable shift during the potential scan (Figure S3B).

The CVA at 288 nm (Figure 2B) shows similar behavior for both optical pathways in the anodic scan. However, in the backward cathodic scan, marked differences were observed, such as the shape and potential at which the absorbance started to decrease due to the regeneration of FcMeOH in solution (+0.17 V for the short path length and +0.14 V for the long path length). These discrepancies are counterintuitive because the shape of the absorption spectra should not be affected by the optical pathway; therefore, both the spectra and their behavior should be similar. These differences can be explained by analyzing the spectral signals in terms of light intensity, specifically the number of counts recorded by the detector rather than the absorbance values. (Figure S4A,B). The spectrum at the long optical path length at the starting potential (0.00 V, blue line in Figure S4B) shows that measurable intensity values are reached from 270 nm onward; below this wavelength, the light intensity is lower than 500–800 counts, close to the limit for obtaining a correct measurement in this spectrometer. It should be noted that this threshold value depends on the spectrometer specifications. This indicates that at wavelengths shorter than 270 nm, the light intensity reaching the detector is too low to be reliable and is highly influenced by the electrical noise of the detector. Conversely, the same spectrum registered at a short optical path length (0.00 V, red line in Figure S4B) displays perfectly measurable intensity values above the spectrometer threshold across the entire UV/vis spectral region.

By studying the evolution of the spectra during the potential scan (Figure S4B), it can be concluded that as FcMeOH oxidation occurs, the UV light intensity in the longest optical pathway becomes insufficient to be measured, providing unreliable information below 300 nm. Furthermore, for the potential of +0.15 V in the reverse scan, no measurable spectral changes were observed between 200 and 300 nm (dotted blue line, Figure S4B). Consequently, the lack of light intensity makes it impossible to calculate the absorbance, rendering Lambert–Beer law inapplicable; therefore, the absorption bands observed in the long optical pathway at potentials where the light intensity is below 500–800 counts must be considered as false bands.

These results also indicate that, in the FcMeOH oxidation process, the evolution of absorbance in the UV region at the shortest optical path length is correct and perfectly correlated with the charge transfer process. Conversely, in the same region, absorbance spectra registered at a long optical path length are only informative at potentials where sufficient light reaches the detector (see Figure S4C, where the evolution of light intensity during the potential scan is plotted). Therefore, the behavior at 288 nm is similar for both optical path lengths in the anodic scan but differs significantly in the cathodic scan. The similarity between the CV and DCVA at 255 nm for the shortest optical path length and at 625 nm for the longest optical path length (Figure S5) indicates that the same process is observed in the electrochemical signal and, at these wavelengths, in the spectroscopic signal. The visible band is much higher in the longest pathway, allowing for the acquisition of a high-quality signal. As shown in Figure S5, the DCVA can be well-defined at 625 nm for the longest optical pathway, obtaining results comparable to those obtained for the shortest optical pathway at 255 nm, while an ill-defined derivative signal is obtained for the shortest optical pathway at 625 nm.

### Nitrate Reduction to Ammonia

The electrochemical nitrate reduction reaction to ammonia (NO_3_RR) is an electrocatalytic strategy proposed to reduce nitrate contamination in water sources, such as lakes or underground waters, by converting this compound into ammonia. This strategy aims simultaneously to reduce nitrate concentration, a problematic pollutant strongly associated with eutrophication,^17,18^ and to generate ammonia, a high-value product for several industrial processes.^19^

NO_3_RR is a highly productive field in which many authors have studied different electrocatalysts to carry this process.^20–23^ Nevertheless, there are few examples in the literature that effectively characterize reaction products using *operando* methodologies. Our group recently reported the use of bidimensional UV/vis-SEC for product characterization during NO_3_RR,^24^ which will be used to demonstrate the advantages of multiscale UV/vis-SEC in this section.

The Cu_2_O electrocatalyst was prepared in a two-step process as described in the literature,^25^ involving electrochemical modification followed by chemical treatment of a Cu disk (full description is provided in the SI). After modifying the Cu-WE surface, NO_3_RR experiments were conducted in a solution 0.075 M KNO_3_ and 1 M KOH. Linear sweep voltammetry (LSV) was performed from +0.40 V to −0.60 at 0.02 Vs^–1^, providing information about the reactant and the products generated between +0.40 V and −0.40 V (Figure 3E). As previously stated in literature,^24,25^ two reduction peaks can be observed in the LSV (green line, Figure 3E,F). The first cathodic peak at +0.10 V was correlated with the reduction of nitrate to nitrite, while the second cathodic peak at −0.14 V was connected to the nitrite reduction to other nitrogen products with a lower oxidation state.

**Figure 3:**
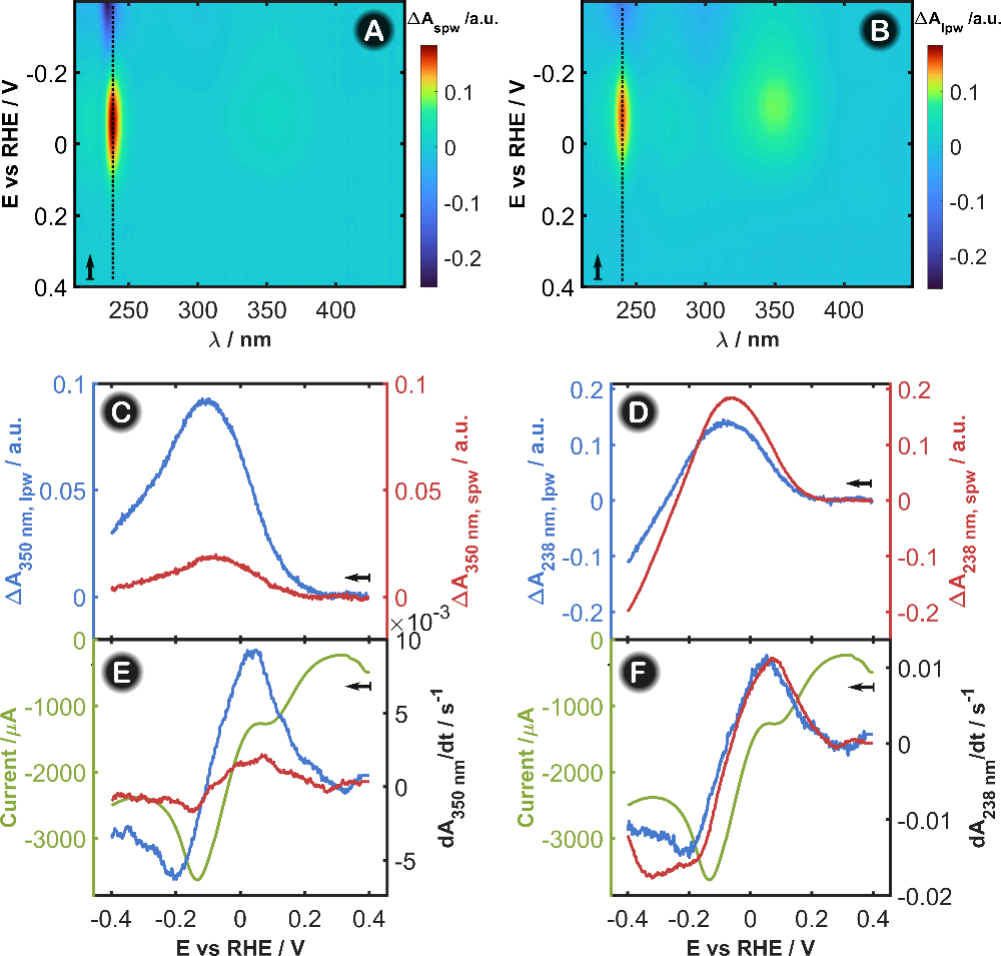
Contour plots displaying the spectra evolution in the (A) short and (B) long optical path lengths. (C) LVAs at 350 nm for long (blue line) and short (red line) optical pathways. (D) LVAs at 238 nm for long (blue line) and short (red line) optical pathways. (E) LSV (green line) and DLVAs at 350 nm for long (blue line) and short (red line) optical pathways. (F) LSV (green line) and DLVAs at 238 nm for long (blue line) and short (red line) optical pathways. Experimental conditions: 0.075 M KNO_3_ solution in 1 M KOH, LSV from +0.40 V to −0.40 at 0.02 V·s^–1^.

Figure 3A,B shows the contour plots representing the evolution of the absorption spectra with the applied potential at the two optical path lengths. In the region between 200 and 400 nm, there are two absorption bands, centered at 300 and 350 nm, related to nitrate consumption and nitrite generation, respectively.^24,26^ In addition, the contour plots also show other absorption bands between 235 and 238 nm (Figure S6A,B). The main difference is that in the shortest optical pathway, the contour plot shows one absorption band at 238 nm that increases to approximately 0.00 V and a second absorption band that decreases at 235 nm at potentials below −0.20 V (Figure S6A), whereas in the longest optical pathway, a unique absorption band evolves, peaking at 238 nm (Figure S6B). As explained previously for FcMeOH, these differences between the two optical path lengths can be related to the light intensity measured in the detector of the spectrometer. For the longest optical pathway, there was sufficient light across the entire spectrum, except below 240 nm (Figure S6C, blue lines). Therefore, considering what was explained in the previous section, false bands could appear when the light intensity is too low. Therefore, in this experiment, it was more reliable to use the shortest optical pathway in the UV spectral region (Figure S6C, red lines).

This behavior significantly affected the absorbance signals registered in the UV/vis-SEC experiments. The linear sweep voltabsorptograms (LVAs) at 350 nm shown in Figure 3C correspond to the spectral region where the light intensity is sufficiently high for the two optical path lengths. The relationship between the maximum absorbance value at −0.10 V for the longest and shortest optical path lengths (A_lpw_/A_spw_) is 5, which is very close to the theoretical value of 4.8, considering the lengths of both optical pathways (d_lpw_/d_spw_). However, the ratio A_lpw_/A_spw_ at 238 nm at −0.10 V (Figure 3D) is 0.75, which is significantly lower than expected. It should be highlighted that the recorded absorbance value was higher for the shortest optical pathway than for the longest pathway. This unreasonable result demonstrates the magnitude of the errors that can occur when the amount of light in the experiment is not considered in the calculations.

The analysis of the derivative of absorbance with respect to time (DLVA) at these two characteristics wavelengths indicates that at 350 nm (blue and red lines, Figure 3E), the behavior is similar to that of the LSV, but with a higher sensitivity in the longest optical path length. Moreover, the DLVA at 350 nm with the shortest optical path length (red line, Figure 3E) provides a response of very poor quality, which is not useful for extracting reliable information. It should be noted that at 238 nm (Figure 3F), although the behavior is similar, the values do not match the expected results because the DLVAs are fully overlapped at the two optical path lengths.

Multiscale UV/vis-SEC has demonstrated that erroneous conclusions may be drawn from absorbance changes in the UV/vis spectral region if the incorrect optical pathway is selected, as the quality of the spectral signal compromises the results obtained during the experiment. Furthermore, it is crucial to note that the most suitable optical pathway can change depending on the spectral region being analyzed. As demonstrated in the NO_3_RR SEC experiments, the most suitable and informative optical pathway below 250 nm is the shortest pathway. However, at wavelengths longer than 250 nm, the longest optical pathway will supply the most sensitive and informative information, as the S/N ratio is higher than that of the shortest optical pathway.

## Conclusions

Multiscale UV/vis absorption SEC is presented in this work. This new experimental approach for SEC measurements was proposed to solve problems in SEC when the ε_λ_ of the compounds involved in the electrode process present different values in the UV and visible regions, or even when products of the reaction are generated at a very low concentration. This new technique is based on the simultaneous measurement of two spectroscopic measurements in a parallel configuration with two different optical pathways. Selecting a suitable scale for measuring absorbance is crucial for obtaining reliable information because, in some cases, the absorbance can be so high that it yields fake absorption bands that are not quantitatively related to the electrochemical reaction. Moreover, this work highlights the importance of considering the amount of light reaching the detector, which is sometimes not sufficiently high to provide accurate information about the reactions occurring at the electrode/solution interface.

Good SEC measurements provide quantitative information about the reactants and/or products involved in the electrode process.^16,24^ The selection of the appropriate optical path length is fundamental to obtain reliable results. For this reason, the user needs to observe the raw spectra of the light source, avoiding to measure in the spectral region where non representative light intensity reaches the spectrometer, which depends on the characteristics of the optical system, including the path length and the detector. Multiscale UV/vis-SEC can be used to study electrochemical reaction in many different fields of chemistry such as, energy, analytical chemistry, materials science, biochemistry, etc.

## Supplementary Material



## Notes

Multiscale instrumental setup and UV/vis SEC instrumentation; multiscale UV/vis SEC cell; comparison of UV/vis-SEC measurements in short and long optical pathway for FcMeOH oxidation process; derivative voltabsorptograms at 625 nm and cyclic voltammogram of FcMeOH oxidation process; synthesis of Cu_2_O catalyst; UV/vis SEC responses at wavelengths lower than 250 nm during nitrate reduction to ammonia; STL documents to fabricate the cell are available upon request from the authors (PDF)
