# Supplementary material for: Multiscale Ultraviolet/Visible Absorption Spectroelectrochemistry in Parallel Configuration
Source: Anal Chem. 2026 Jun 19;98(25):18326–31. doi: 10.1021/acs.analchem.6c01563 (PMC13325444; doi:10.1021/acs.analchem.6c01563)
Supplement: Supplementary file 1 [file ac6c01563_si_001.pdf]

Supporting Information

Multiscale UV/vis Absorption Spectroelectrochemistry in Parallel Configuration

Maria Huidobro, Martin Perez-Estebanez, Aranzazu Heras, Alvaro Colina**

Department of Chemistry, Universidad de Burgos, Pza. Misael Bañuelos s/n, E-09001, Burgos, Spain.

*Corresponding autors: Alvaro Colina, acolina@ubu.es; Aránzazu Heras, maheras@ubu.es

■ INDEX

1. **Multiscale instrumental setup and UV/vis-SEC instrumentation 1**
2. **Multiscale UV/vis-SEC cell 2**
3. **Comparison of UV/vis-SEC measurements in short and long optical pathway for FcMeOH oxidation process 3**
4. **Derivative voltabsorptograms at 625 nm and cyclic voltammogram of FcMeOH oxidation process 7**
5. **Synthesis of Cu_2O catalyst 9**
6. **UV/vis-SEC responses at wavelengths lower than 250 nm during nitrate reduction to ammonia........ 10**
7. **References 12**

1. Multiscale instrumental setup and UV/vis-SEC instrumentation

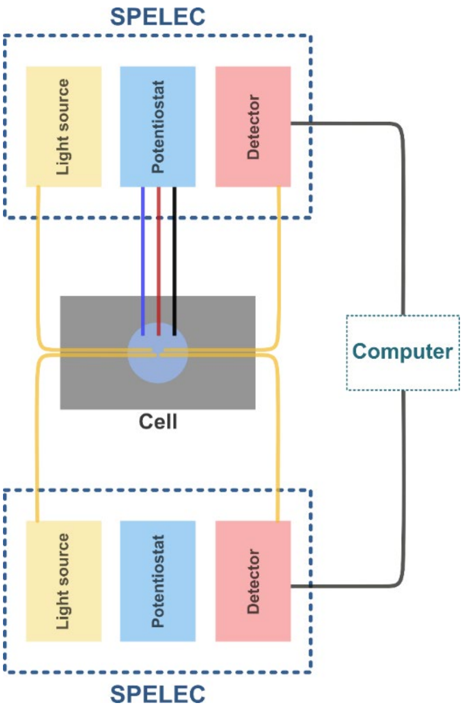

Figure S1. Schematic representation of the multiscale UV/vis-SEC instrumental setup.

UV/vis-SEC experiments were performed using two customized SPELEC instruments (Metrohm-DropSens). Each SPELEC instrument contains a potentiostat, a UV/vis light source, and a photodiode-array spectrometer with a spectral range of 210–990 nm. One of the SPELEC instruments was used to control the electrode process with a potentiostat and to measure the evolution of the UV/vis absorption spectra in one of the two optical path lengths. The other SPELEC instrument was only used to simultaneously register the evolution of the UV/vis absorption spectra between 210 and 990 nm in the second optical path length. In the spectrophotometric measurements two halogen-deuterium light sources connected to two 100 μm optical fibers (Ocean Optics) were used to guide the electromagnetic radiation from the light source to the multiscale UV/vis-SEC cell. Two additional 100 μm bare optical fibers (Ocean Optics) collected the transmitted light from the two optical path lengths to the two spectrophotometers.

2. Multiscale UV/vis-SEC cell

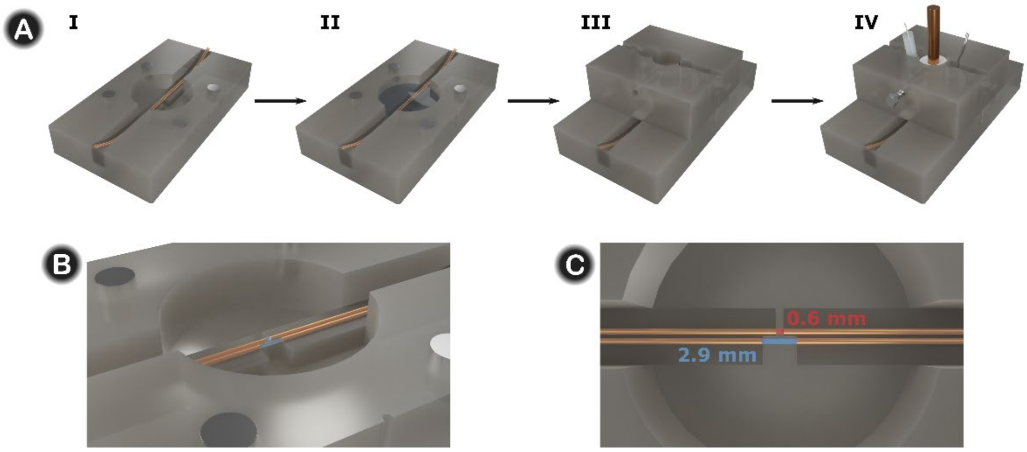

Figure S2. (A) Schematic of the cell assembly used for the UV/vis-SEC multiscale experiments. (I) Bottom body. (II) The solution is added to the cell. (III) The lid or upper body is attached and held in place with magnets. (IV) The electrodes are placed on top of the working electrode and secured with a screw. (B) Zoom of the pool with optical fibers at both path lengths. (C) Schematic of optical fiber positioning. Light was conducted in the opposite direction in each pair of optical fibers to avoid optical interference.

3. Comparison of UV/vis-SEC measurements in short and long optical pathway for FcMeOH oxidation process

Figure S3A shows the spectra at the vertex potential (+0.25 V) for the experiment depicted in Figure 2 in the manuscript. In the visible region of the spectra, there are significant similarities. In both optical pathways one absorption band emerges at 625 nm (Figure S3A). The evolution of the absorbance at this wavelength with the applied potential (cyclic voltabsorptogram, CVA) shows the same behavior in the two optical pathways and almost overlaps during the potential scan, indicating that the information extracted is related to the same process: oxidation and subsequent reduction of FcMeOH (Figure S3C). These similarities also indicate that sufficient light intensity is recorded in this spectral range in the two optical pathways (Figure S4A). However, the longest path length exhibits greater sensitivity and, therefore, much less noise and a better S/N ratio than the short path length (Figure S3C). The similarity between the A_{lpw}/A_{spw} and d_{lpw}/d_{spw} ratios is consistent with the Lambert-Beer law, which dictates that absorbance is proportional to the optical path length.

Nevertheless, a significant difference was observed in the spectral region between 200 and 400 nm (Figure S3A). In this UV spectral region (inset 1, Figure S3A), the spectrum of the shortest optical path length displays two overlapping bands (288 and 255 nm), whereas in the spectrum of the long path length, only one absorption band can be seen that shifts between 290 and 282 nm during the CV (Figure S3B). The absorption band at 255 nm was not observed in the long optical pathway because the light intensity was too low to observe changes in absorbance during the potential scan (blue lines in Figure S4B and blue dotted line in Figure S4C). The light count values were below the threshold of 500-800 counts, indicating a lack of reliability in the recorded light intensity values (dashed gray line, Figure S4B). Conversely, in the short optical pathway, the intensity of

the light reaching the detector exceeded the threshold value, thereby enabling the clear observation of all spectral changes in this area (red lines in Figure S4B and red dotted line in Figure S4C).

The second absorption band in the UV region at 288 nm showed reliable behavior for the shortest path length because the intensity of light registered was higher than the threshold of 500-800 counts during the entire experiment (red lines in Figure S4B and red solid line in Figure S4C). For the longest optical path length, only up to a potential of approximately +0.20 V during the anodic scan, the amount of light recorded by the detector exceeded the threshold value (blue solid line in Figure S4C). This can be clearly observed in Figure S4B, where the intensities of the spectra registered at the starting potential and at +0.15 V in the backward scan are plotted (dotted lines in Figure S4B); the spectral region at which insufficient light reaches the detector increases during the oxidation of FcMeOH. For this reason, the CVA at 288 shows significant differences from that recorded for the shortest optical path length (Figure 2C).

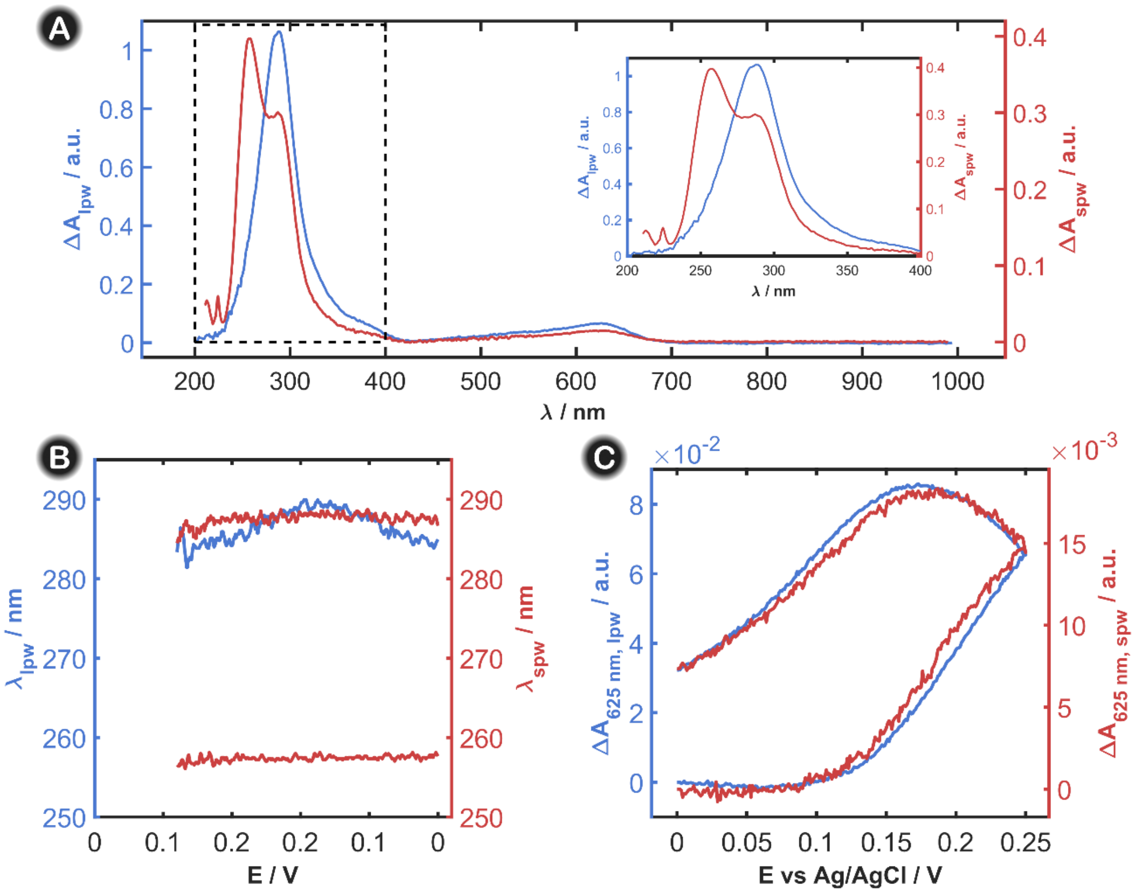

Figure S3. (A) UV/vis absorption spectra at the vertex potential (+0.25 V) for long (blue line) and short (red line) optical pathway. (B) Evolution of maximum absorbance at each potential in the spectral region between 250 and 295 nm. (C) CVAs at 625 nm for long (blue line) and short (red line) optical pathways. Experimental conditions: 2.5 mM FcMeOH solution in 0.1 M KCl, CV from 0 V to +0.25 V at 0.02 $V \cdot s^{-1}$.

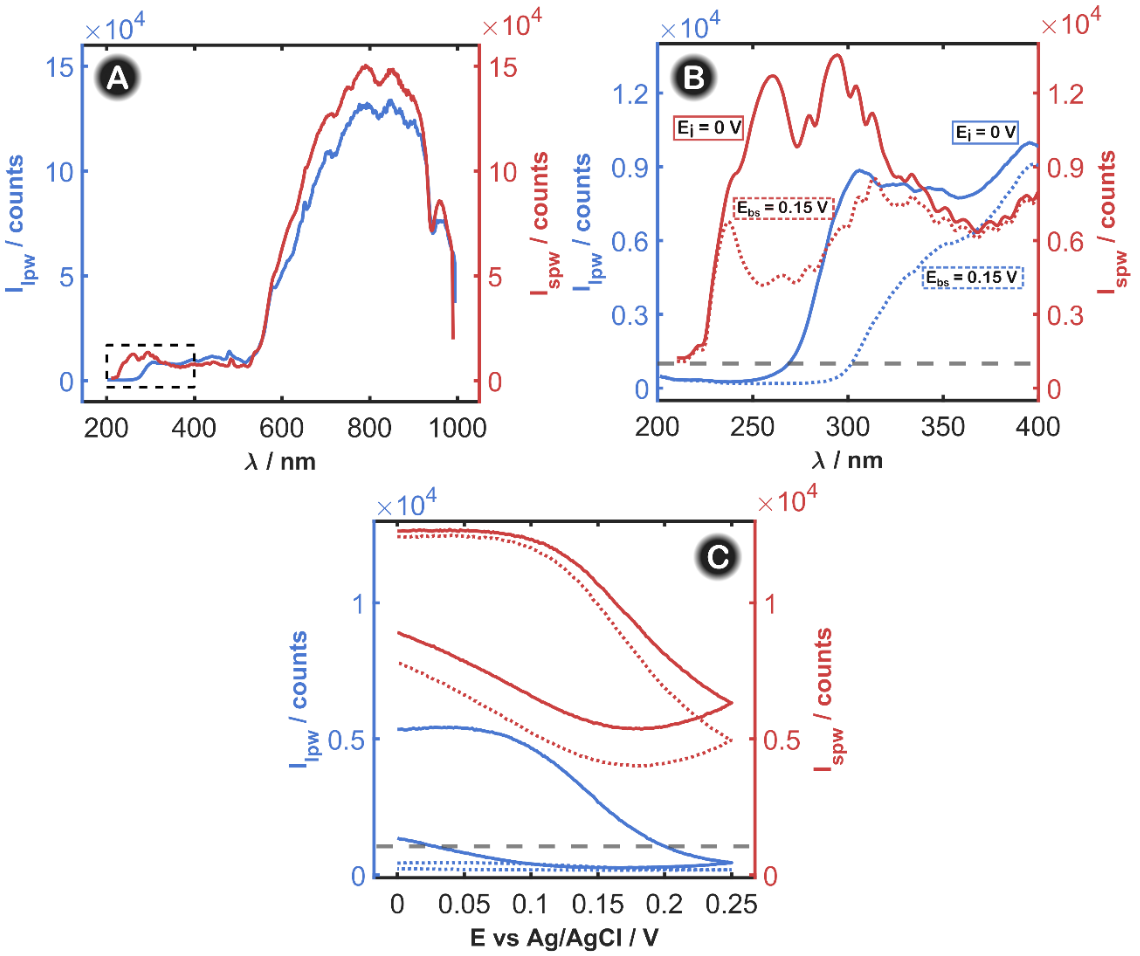

Figure S4. (A) Intensity spectra at the starting potential (0 V) for long (blue line) and short (red line) optical pathways. (B) Zoom of intensity spectra between 200 and 400 nm at the starting potential (0 V, solid lines) and at +0.15 V in the backward scan (dotted lines) for long (blue lines) and short (red lines) optical pathways. (C) Evolution of the intensity of the spectra at 288 nm (solid lines) and 255 nm (dotted lines) with the potential for the longest (blue lines) and shortest (red lines) optical path lengths. Experimental conditions: 2.5 mM FcMeOH solution in 0.1 M KCl, CV from 0 V to +0.25 V at 0.02 $V \cdot s^{-1}$.

4. Derivative voltabsorptograms at 625 nm and cyclic voltammogram of FcMeOH oxidation process

Figure S5 represents the CV (green line) of a 2.5 mM FcMeOH solution in 0.1 M KCl between the vertex potentials 0 V and +0.25 V, starting at 0 V in the anodic direction at 0.02 $V \cdot s^{-1}$ compared to different derivatives of the absorbance with respect to time because the derivative cyclic voltabsorptograms (DCVA) at a specific wavelength is clearly related to the CV. Figure S5A shows the DCVA at 255 nm for a short optical path length (red line). The band at 255 nm for the shortest optical path length, which is suitable for visualizing the behavior in the UV region, showed a derivative very similar to the CV. Figure S5B shows the DCVA at 255 nm for the longest optical path length; in this case, the derivative is not related to the CV because there is not enough light at 255 nm, as shown in Figure S4B (blue lines). Figure S5D shows the DCVA at 625 nm for the longest optical path length (blue line), where the same behavior between the two signals is clearly observed, confirming the conclusions obtained from Figure S3C. In the case of the DCVA at 625 nm for the shortest optical pathway (red line, Figure S5C), the signal-to-noise (S/N) ratio is lower than that of the signal plotted in Figure S5A, where higher values of absorbance are registered; therefore the quality of the signal is very poor, although similarities between the CV and DCVA can be observed.

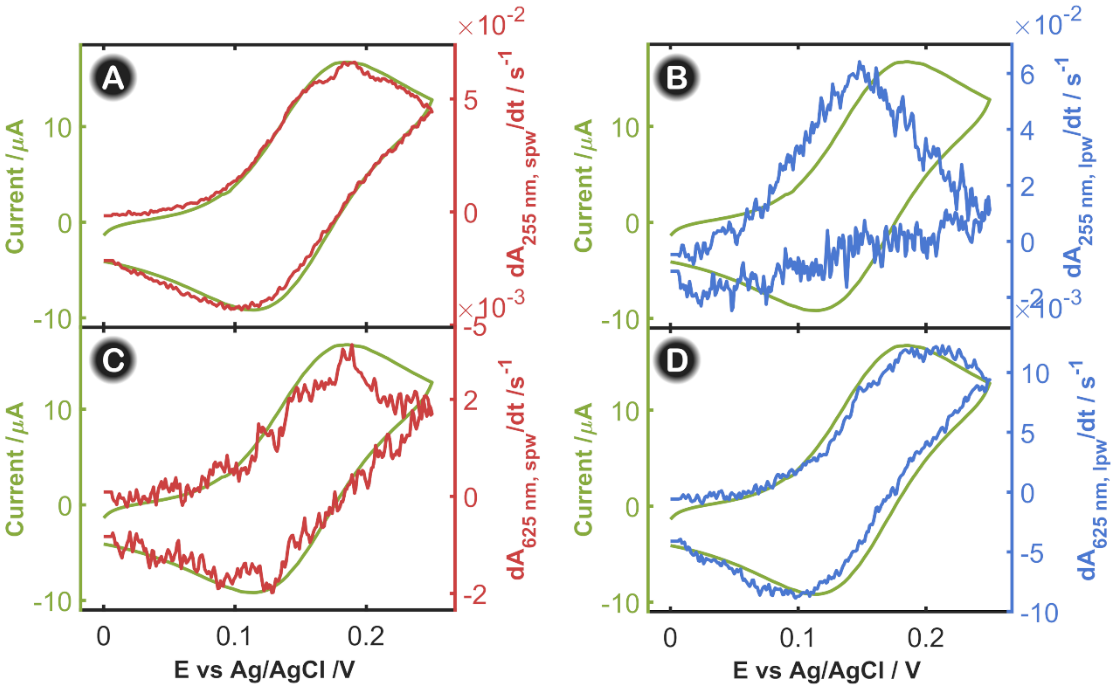

Figure S5. CV (green line) compared with (A) DCVA at 255 nm (red line) for the shortest optical pathway, (B) DCVA at 255 nm (blue line) for the longest optical pathway, (C) DCVA at 625 nm for the shortest optical pathway (red line), and (D) DCVA at 625 nm (blue line) for the longest optical pathway. Experimental conditions: 2.5 mM FcMeOH solution in 0.1 M KCl, CV from 0 V to +0.25 V at 0.02 $V \cdot s^{-1}$.

5. Synthesis of Cu_2O catalyst

Cu_2O electrocatalyst was prepared through a two-step process: electrochemical modification followed by chemical treatment of a Cu disk. Before carrying out the modification, the Cu disk electrode was thoroughly polished with alumina 0.05 µm to obtain a mirror-like finish, and subsequently, it was sonicated in deionized water to clean its surface. The first step consisted of an electrochemical oxidation of the Cu electrode by applying +1.10 V *vs* Ag/AgCl in 0.1 M KCl for 100 s.[1] Subsequently, the electrode was rinsed with deionized water and subjected to the second step. This consisted of a chemical treatment by immersing the electrode in a solution of 0.1 M $NaHCO_3$ for 10 min. After this stage, a Cu_2O-based electrode composed of superficial nanocubes was obtained and used for NO_3RR. The characterization of the Cu_2O substrate was performed in previous works.[2]

6. UV/vis-SEC responses at wavelengths lower than 250 nm during nitrate reduction to ammonia.

The contour plots in the spectral region between 220 and 250 nm show different behaviors for the two optical path lengths (Figures S6A and S6B). For the shortest optical path length (Figure S6A), two different absorption bands were observed at 238 and 235 nm. Nevertheless, for the longest optical path length (Figure S6B), only one band around 240 nm was observed, with a behavior like that depicted for the shortest path length but shifted to longer wavelengths. As discussed in the main text, the UV/vis absorption spectra should not change depending on the optical path length chosen, and discrepancies between the observed spectra in the shortest and longest optical pathways can be rationalized by observing the light quantity of the measurements in each configuration.

An in-depth analysis of the spectra recorded in terms of light intensity at the initial potential (+0.40 V) and final potential (-0.40 V), as illustrated in Figure S6C, reveals that the spectral region between 220 and 250 nm lacks sufficient light to ensure the trueness of the absorption bands in this region of the spectrum for the longest optical path length. However, for the shortest optical path length, the light intensity recorded between 225 and 240 nm exceeded the threshold value of 500-800 counts, as indicated by the experiments with FcMeOH. For the absorption bands between 260 and 400 nm, sufficient light intensity was present in both optical path lengths (inset, Figure S6C), ensuring the reliability of the spectroscopic information extracted from the evolution with the potential of the UV/vis absorbance spectra.

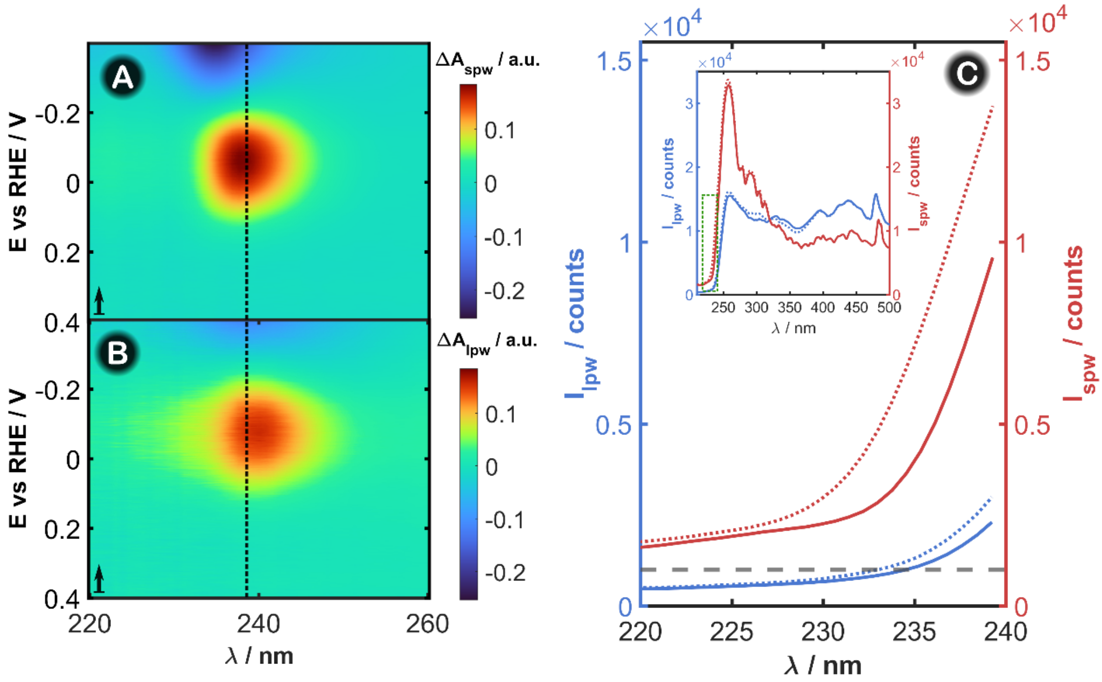

Figure S6. Contour plots displaying the spectra evolution between 220 and 260 nm, of a in the shortest (A) and longest (B) optical path lengths. (C) Intensity spectra at +0.40 V (starting potential, solid line) and -0.40 V (end potential, dotted line) for the longest (blue line) and shortest (red line) optical path length; inset shows the intensity spectra between 225 and 500 nm. Experimental conditions: 0.075 M KNO_3 solution in 1 M KOH, LSV from +0.40 V to -0.40 V at 0.02 V s^{-1}.

7. References

(1) Perales-Rondon, J. V.; Rojas, D.; Gao, W.; Pumera, M. Copper 3D-Printed Electrodes for Ammonia Electrosynthesis via Nitrate Reduction. *ACS Sustain. Chem. Eng.* **2023**, *11* (18), 6923–6931. https://doi.org/10.1021/acssuschemeng.2c06851.

(2) Huidobro, M.; Romay, L.; Perez-Estebanez, M.; Heras, A.; Perales-Rondon, J. V.; Colina, A. Operando UV/Vis Absorption Spectroscopy for Studying the Nitrate to Ammonia Conversion on Cu_2O-Based Electrodes. *ACS Catal.* **2026**, 16, 2331-2339 https://doi.org/10.1021/acscatal.5c07326.
